# Correlations between cognitive reserve, gray matter, and cerebrospinal fluid volume in healthy elders and mild cognitive impairment patients

**DOI:** 10.3389/fneur.2024.1355546

**Published:** 2024-03-01

**Authors:** Liang Zhou, Wenxia Yang, Yang Liu, Jiachen Li, Mengmeng Zhao, Guangyao Liu, Jing Zhang

**Affiliations:** ^1^Department of Magnetic Resonance, The Second Hospital of Lanzhou University, Lanzhou, China; ^2^Second Clinical Medical School, Lanzhou University, Lanzhou, China; ^3^Gansu Province Clinical Research Center for Functional and Molecular Imaging, Lanzhou, China; ^4^Shanghai United Imaging Intelligence, Shanghai, China

**Keywords:** cognitive reserve, gray volume, cognitive function, cerebrospinal fluid, mild cognitive impairment

## Abstract

**Objective:**

To explore the effect of cognitive reserve (CR) on brain volume and cerebrospinal fluid (CSF) in patients with mild cognitive impairment (MCI) and healthy elders (HE).

**Methods:**

31 HE and 50 MCI patients were collected in this study to obtain structural MRI, cognitive function, and composite CR scores. Educational attainment, leisure time, and working activity ratings from two groups were used to generate cognitive reserve index questionnaire (CRIq) scores. The different volumes of brain regions and CSF were obtained using uAI research portal in both groups, which were taken as the regions of interest (ROI), the correlation analysis between ROIs and CRIq scores were conducted.

**Results:**

The scores of CRIq, CRIq-leisure time, and CRIq-education in HE group were significantly higher than patients in MCI group, and the montreal cognitive assessment (MoCA) and minimum mental state examination (MMSE) scores were positively correlated with the CRIq, CRIq-education in both groups, and were positively correlated with CRIq-leisure time in MCI group. The scores of auditory verbal learning test (AVLT) and verbal fluency test (VFT) were also positively correlated with CRIq, CRIq-leisure time, and CRIq-education in MCI group, but the score of AVLT was only positively correlated with CRIq in HE group. Moreover, in MCI group, the volume of the right middle cingulate cortex and the right parahippocampal gyrus were negatively correlated with the CRIq, and the volume of CSF, peripheral CSF, and third ventricle were positively correlated with the CRIq-leisure time score. The result of mediation analysis suggested that right parahippocampal gryus mediated the main effect of the relationship between CRIq and MoCA score in MCI group.

**Conclusion:**

People with higher CR show better levels of cognitive function, and MCI patients with higher CR showed more severe volume atrophy of the right middle cingulate cortex and the right parahippocampal gyrus, but more CSF at a given level of global cognition.

## Introduction

The concept of cognitive reserve (CR) is proposed to explain the mismatch between pathological changes and functional changes in the brain (1) and is considered to be a potential protective mechanism for the prevention of neurodegenerative diseases such as Alzheimer disease (AD). It can reflect the ability of dynamic changes in brain structure and function (2) and to explain what keeps some people cognitively competent and slows the progression of dementia. The pattern of brain activation is opposite between older individuals with normal aging and pathological aging under the modulation of CR (3), and the variances in the efficacy of brain neural networks and the application of neural networks may be one of the mechanisms by which CR provides protection against brain aging or AD pathology (4). Several studies have showed that at specific cognitive levels, subjects with higher CR levels have higher levels of Aβ42 in cerebrospinal fluid (CSF) (5), worse fiber bundle integrity (6), and lower cerebral metabolism and perfusion (7, 8) compared with subjects with lower CR levels, indicating that subjects with higher CR levels can tolerate more severe brain pathological changes and maintain the same cognitive performance as those with lower CR levels in response to milder pathological changes.

Mild cognitive impairment (MCI) is considered to be a symptomatic stage that occurs between normal aging and dementia. It has been shown that MCI patients with higher levels of CR are significantly more likely to reverse to normal cognition than progress to dementia (9) and high CR is correlated with a lower risk of MCI as well as a positive role in alleviating cognitive decline (10–12). Neuroimaging studies have also confirmed that CR is associated with increased connectivity in cognitive control networks as well as between the left frontal cortex and the dorsal attentional network in MCI patients (13, 14). What is more, CR has also been shown to modulate the cortical architecture, white matter macromolecular volume, and cerebral blood flow to be protective against the cognitive impairment effect in MCI patients (15–17). It has been found that MCI patients with higher CR are able to tolerate greater brain pathology such as cortical thinning or gray matter volume atrophy as well as having more severe white matter damage than patients with lower CR at a given level of cognitive impairment (18). Recent evidences have suggested that education level and higher occupational status significantly affected the relationships between hippocampal volume and executive control function, total gray matter volume and language function in MCI patients (19).

Cerebrospinal fluid circulation includes not only the directed flow of cerebrospinal fluid but also pulsating movements throughout the brain, as well as the local fluid exchange between interstitial fluid and cerebrospinal fluid in the Virchow-Robin space. This rapid and sustained two-way fluid exchange is essential for the brain to remove wasted molecules, such as the A*β* and tau protein (20), which are clearly associated with cognitive impairment in AD patients. Larger choroid plexus volume is correlated with severity of cognitive decline in MCI patients, and the choroid plexus is associated with cerebrospinal fluid production (21). The volume of CSF is independently correlated with cognitive function, and may be crucial early indicators of risk for cognitive impairment in young individuals (22). The education level is the most widely used as a proxy for CR in present studies and it is the most closely related to dementia (23), but it is well known that CR is not only affected by educational attainment, but also by occupational achievement, social, cognitive, and leisure time activity. A longitudinal study demonstrated that well-educated individuals or individuals who are more active in leisure activity have a lower risk of AD (24). It is now well established that improving education level and life skills training can help improve CR, reduce the risk of MCI, and delay the occurrence of clinical symptoms and progression (10). Data from meta-analysis study also suggested that intelligence quotient, occupational attainment, and cognitive activity were all correlated with CR (25). Evidence suggests that education is among the most important factors for CR and most current studies have used educational attainment as a single cognitive proxy or other single cognitive proxy, while fewer have used comprehensive CR with occupational attainment, cognitive activity. Although individual brain MR imaging volumes are significantly correlated with cognitive function, the correlation was also influenced by other known factors such as CR, and the relationship between CSF volume and CR is unknown.

Thus, in this study, we use the comprehensive index of education level, work activities and leisure time as proxy of CR, and combined with MRI to explore whether CR can regulate brain and CSF volume and its relationship between cognitive function in MCI patients.

## Materials and methods

### Participants

A total of 81 participants were recruited from memory disorder clinic at the Department of Neurology in Lanzhou University Second Hospital and local community, including 50 MCI and 31HE. The diagnosis of MCI was performed according to current diagnostic criteria (26) and the clinical dementia rating (CDR) score was less than 0.5. The enrollment criteria for HE group were: (a) age greater than 50 years; (b) general physical condition is normal; (c) overall cognitive function is normal, Minimum Mental State Examination (MMSE) score is 27–30 points; (d) CDR score of 0 points; (e) no memory decline; and (f) right-handed. This study was approved by the Ethics Committee of Lanzhou University Second Hospital and informed consent was written by all participants.

### Neuropsychological and cognitive reserve assessment

The Montreal Cognitive Assessment (MoCA) and MMSE were used to measure general cognitive function, memory recall was measured by the Auditory Verbal Learning Test (AVLT, Chinese version), verbal fluency was measured by verbal fluency test (VFT). The cognitive reserve index questionnaire (CRIq) is a questionnaire designed by Nucci et al. that combines educational attainment, occupational attainment, and cognitive activity to evaluate the CR of an individual (27). The CRIq is derived by summing the scores of three components: CRI-education, CRI-working activity, and CRI-leisure time. CRI-education records the years of education. CRI-working activity divided into five categories according to different type, including low skilled manual work, skilled manual work, skilled non-manual work, professionals, and senior intellectuals. It was recorded the working time of each job (5 years as a unit, less than 5 years, then rounded up to 5), multiply the time of work by the sum of the scores of the levels of work (1–5) as the CRI-working activity. CRI-leisure time refers to all of those activities that are normally carried out in spare time. The time*frequency of the activity items in the scale (including reading, driving, sports, etc.) is used as the score for this item. According to the standardized questionnaire, a higher CRI score represents a higher CR for the subject.

### MRI acquisition

All participants underwent MRI-3 T brain scanning (Ingenia CX, Philips Healthcare, the Netherlands) with a 32-channel head coil. The whole-brain T1-weighted images were acquired with following protocol: TR = 5.9 ms, TE = 3.7 ms, flip angle = 8°, FOV = 256 × 256 mm^2^, and voxel size = 1 mm × 1 mm × 1 mm. A high-resolution 3D T2 weighted image was also performed to exclude brain disorders (e.g., strokes, tumor) with following protocol: TR = 3,000 ms, TE = 250 ms, FOV = 256 × 256 mm^2^, and voxel size = 1 mm × 1 mm × 1 mm. The whole brain subregions of both groups were extracted automatically by the uAI research portal (uRP, https://www.uii-ai.com/en/uai/scientific-research). The uRP is a multifunctional platform to perform accurate image processing and analysis (28), which can handle high-resolution MR images and hierarchically segment brain structures to gray matter, white matter, cerebrospinal fluid (CSF), and the medial temporal lobe atrophy (MTA) score. The processing pipeline involved automatic parcellation of the structural MRI data using a 3D deep learning segmentation model, which achieved a Dice similarity coefficient of 91.06% between the automatically segmented results and ground truth data. Note that the ground truth is initially obtained from FreeSurfer software and then further refined by an experienced rater based on the Desikan-Killiany atlas (29, 30). The workflow mainly includes: (a) the bias field correction, (b) removal of the skull, (c) tissue segmentation of white matter, gray matter, and cerebrospinal fluid (CSF), (d) bilateral segmentation, and (e) parcellation of 109 sub-regions. Following the brain parcellation step, brain volumes were automatically calculated. The automatic image segmentation module on the uRP has been used in relationship between AD and the whole brain subregions based on structural MRI (31). The difference volume of brain regions, CSF and MTA scores between two groups were extracted.

### Statistical analyses

Data with normal distribution are expressed as mean (standard deviation, SD), and the independent sample *t*-test is used for comparison between groups. Data with non-normal distribution are expressed as median (interquartile range), and the Mann–Whitney U test is used for between-group comparisons. Statistical analysis was performed in SPSS22 software, and two groups of brain regions with volume differences were extracted and correlated with CRIq for analysis. Both groups obeyed normal distribution with Pearson correlation analysis, otherwise Spearman correlation analysis was used, and the difference was considered statistically significant at *p* < 0.05.

## Results

### Participant characteristics

As shown in Table 1, we observed the scores of CRIq, CRIq-education, and CRIq-leisure time and cognitive scores in NC group were significantly higher than MCI patients. Age, Sex, CRIq-work activity, ADL, IADL, and GDS did not differ significantly between the two groups. Table 2 showed the significantly different of MTA, CSF, and different volumes of brain regions between the two groups. As showed in Table 2, the volume of right parahippocampal gyrus, left middle temporal gyrus, bilateral lateral occipital gyrus, bilateral nucleus accumbens, mid-anterior corpus callosum, mid-corpus callosum, bilateral hippocampus, left thalamus, right middle cingulate cortex, left posterior cingulate cortex, bilateral hippocampus presubiculum, left dentate gyrus, and bilateral hippocampus fimbria in HE group were higher than patients in MCI group, but the left MTA score, the volume of CSF, peripheral CSF, right choroid plexus, third ventricle, and fourth ventricle were higher in MCI group than HE group.

**Table 1:** Principal demographic and clinical characteristics of the participants.

	HE (*n* = 31)	MCI (*n* = 50)	*p*
Age (years)	59.48 (7.04)	63.5 (59, 68)	0.164
Sex (m/f)	8/20	15/35	0.927
Education (years)	11.32 (2.34)	8.5 (5.75, 11)	<0.001^*^
CRIq	100.61 (13.54)	90 (82, 95)	0.002^*^
CRIq-education	99 (94, 108)	95 (85, 99.25)	0.004^*^
CRIq-work activity	111 (89, 117)	94 (87, 111)	0.114
CRIq-leisure time	91.81 (11.88)	86.38 (9.83)	0.036^*^
MoCA	27 (26, 27)	21 (17.75, 23)	<0.001^*^
MMSE	28 (27, 29)	25 (23, 26)	<0.001^*^
AVLT-delay	9 (9–11)	4 (3, 5)	<0.001^*^
VFT	15 (18–20)	14 (12, 16)	<0.001^*^
ADL	8 (8, 8)	8 (8, 8)	0.688
IADL	12 (12, 12)	12 (12, 13)	0.146
GDS	2 (1, 6)	1 (1, 6)	0.478

Data with normal distribution are expressed as mean (SD). Data with non-normal distribution are expressed as median (interquartile range); HE, Healthy elders; MCI, Mild cognitive impairment; CRIq, Cognitive reserve index questionnaire; MMSE, Mini-mental state examination; MoCA, Montreal cognitive assessment; AVLT, Auditory verbal learning Test; VFT, Verbal fluency test; ADL, Activities of daily living; IADL, Instrumental activity of daily living; and GDS, The geriatric depression scale; ^*^p < 0.05.

**Table 2:** The significantly different volumes of brain regions of the participants.

MRI measures	HE (*n* = 31)	MCI (*n* = 50)	*p*
Left MTA	0 (0, 0)	0 (0, 0.25)	0.013
CSF (cm^3^)	302.51 (44.56)	339.14 (62.09)	0.005
Peripheral CSF (cm^3^)	278.99 (39.71)	306.65 (53.88)	0.016
Right parahippocampal gyrus (cm^3^)	1.99 (0.22)	1.88 (0.23)	0.031
Left middle temporal gyrus	10.49 (1.34)	9.87 (1.25)	0.036
Left lateral occipital gyrus (cm^3^)	11.35 (1.64)	10.61 (1.35)	0.029
Right lateral occipital gyrus (cm^3^)	11.15 (1.39)	10.57 (1.13)	0.043
Left nucleus accumbens (cm^3^)	0.37 (0.12)	0.3 (0.12)	0.009
Right nucleus accumbens (cm^3^)	0.48 (0.11)	0.4 (0.94)	0.003
Right choroid plexus (cm^3^)	0.69 (0.2)	0.85 (0.25)	0.003
Mid-anterior corpus callosum (cm^3^)	0.63 (0.15)	0.52 (0.11)	<0.001
Mid-corpus callosum (cm^3^)	0.58 (0.15)	0.51 (0.11)	0.032
Left hippocampus (cm^3^)	4.21 (3.96, 4.34)	4 (3.7, 4.3)	0.033
Right hippocampus (cm^3^)	4.36 (0.46)	4.09 (3.87, 4.4)	0.036
Left thalamus (cm^3^)	7 (0.58)	6.61 (6.2, 7.2)	0.036
Right middle cingulate cortex (cm^3^)	1.9 (1.71, 2.08)	1.73 (1.56, 2.04)	0.038
Left posterior cingulate cortex (cm^3^)	3.13 (0.43)	2.8 (2.63, 3.06)	0.005
Left hippocampus presubiculum (cm^3^)	0.3 (0.03)	0.28 (0.04)	0.015
Right hippocampus presubiculum (cm^3^)	0.27 (0.03)	0.26 (0.03)	0.048
Left dentate gyrus (cm^3^)	0.3 (0. 03)	0.27 (0.26, 0.3)	0.014
Left hippocampus fimbria (cm^3^)	0.53 (0.05)	0.49 (0.46, 0.53)	0.019
Right hippocampus fimbria (cm^3^)	0.56 (0.07)	0.52 (0.49, 0.57)	0.016
Third ventricle (cm^3^)	1.26 (0.4)	1.52 (1.15, 1.88)	0.028
Fourth ventricle (cm^3^)	1.48 (1.17, 1.69)	1.56 (1.32, 1.89)	0.039

Data with normal distribution are expressed as mean (SD). Data with non-normal distribution are expressed as median (interquartile range); MTA, Median temporal lobe atrophy; CSF, Cerebrospinal fluid.

### Relationship between CR and cognitive function

**Figure 1:**
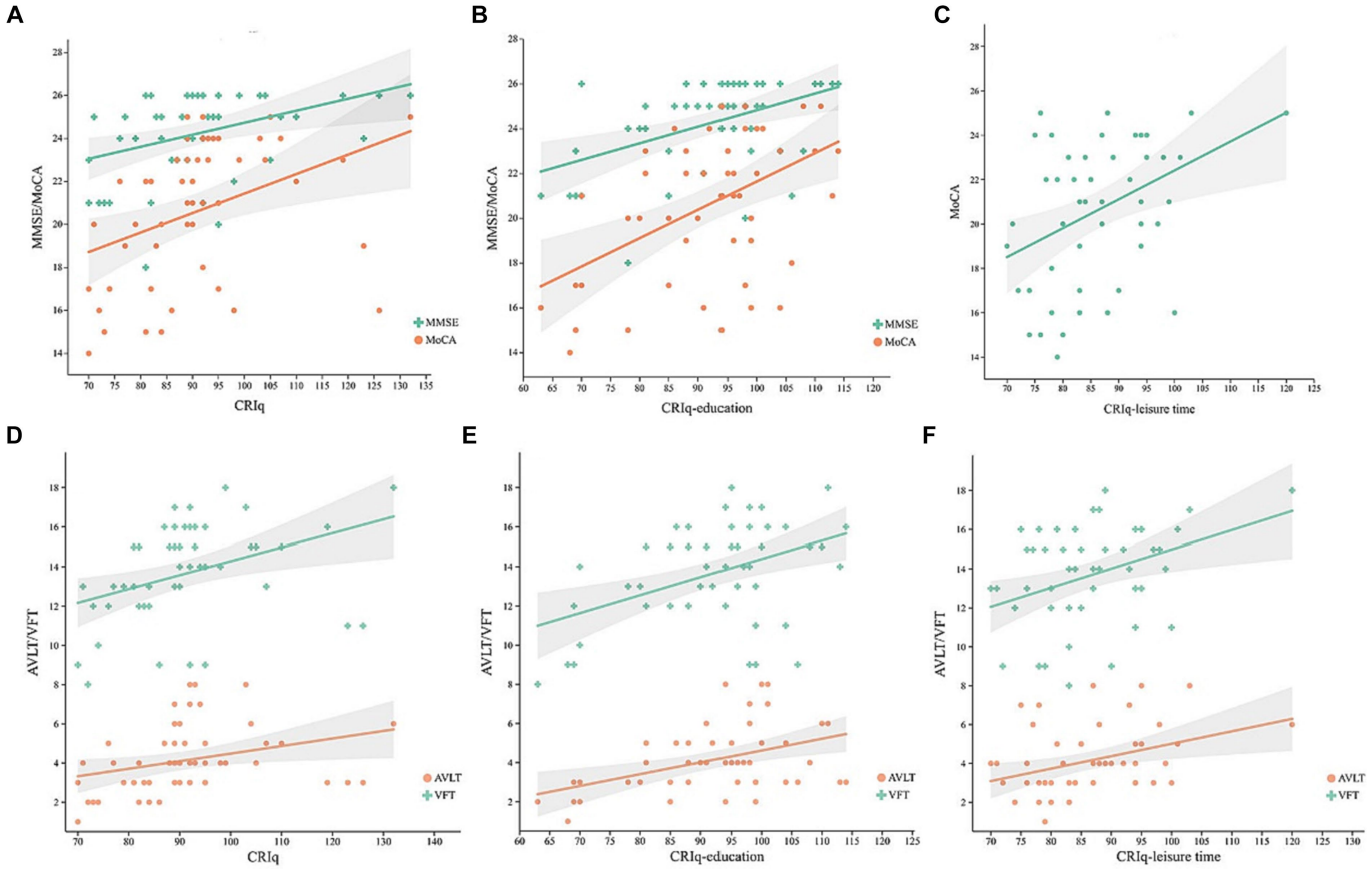
In MCI group, scatterplots of MoCA and MMSE scores with the CRIq **(A)**, CRIq-education **(B)**, CRIq-leisure time **(C)**, and scatterplots of AVLT and VFT scores with the CRIq **(D)**, CRIq-education **(E)**, and CRIq-leisure time **(F)** in MCI group.

**Figure 2:**
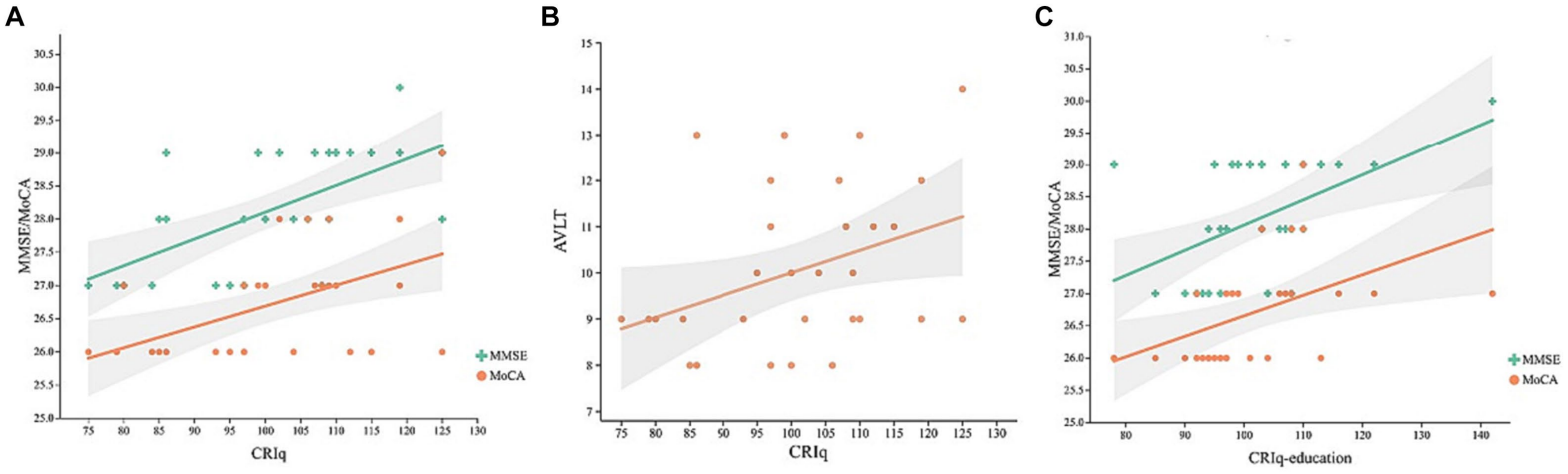
Scatterplots of CRIq with MoCA and MMSE scores **(A)**, AVLT **(B)**, and CRIq-education with MoCA and MMSE scores **(C)** in HE group.

In the MCI group, controlling for age, gender, MoCA, and MMSE were positively correlated with the CRIq (Figure 1A, *r* = 0.408, *p* = 0.003; *r* = 0.49, *p* < 0.001, respectively), CRIq-education (Figure 1B, *r* = 0.387, *p* = 0.006; *r* = 0.437, *p* = 0.002, respectively), and MoCA was also positively correlated with CRIq-leisure time (Figure 1C, *r* = 0.383, *p* = 0.006). Besides, AVLT and VFT were positively correlated with CRIq (Figure 1D, *r* = 0.434, *p* = 0.002; *r* = 0.437, *p* = 0.002, respectively), CRIq-education (Figure 1E, *r* = 0.403, *p* = 0.004; *r* = 0.374, *p* = 0.007, respectively) and CRIq-leisure time (Figure 1F, *r* = 0.383, *p* = 0.006; *r* = 0.35, *p* = 0.013, respectively). In the NC group, controlling for age, gender, and CRIq were positively correlated with MoCA (Figure 2A, *r* = 0.498, *p* = 0.004), MMSE (Figure 2A, *r* = 0.664, *p* < 0.001), AVLT (Figure 2B, *r* = 0.38, *p* = 0.035), CRIq-education was positively correlated with MoCA and MMSE (Figure 2C, *r* = 0.626, *p <* 0.001; *r* = 0.518, *p* = 0.003, respectively).

### Relationship between CRIq and brain volume in MCI group

**Figure 3:**
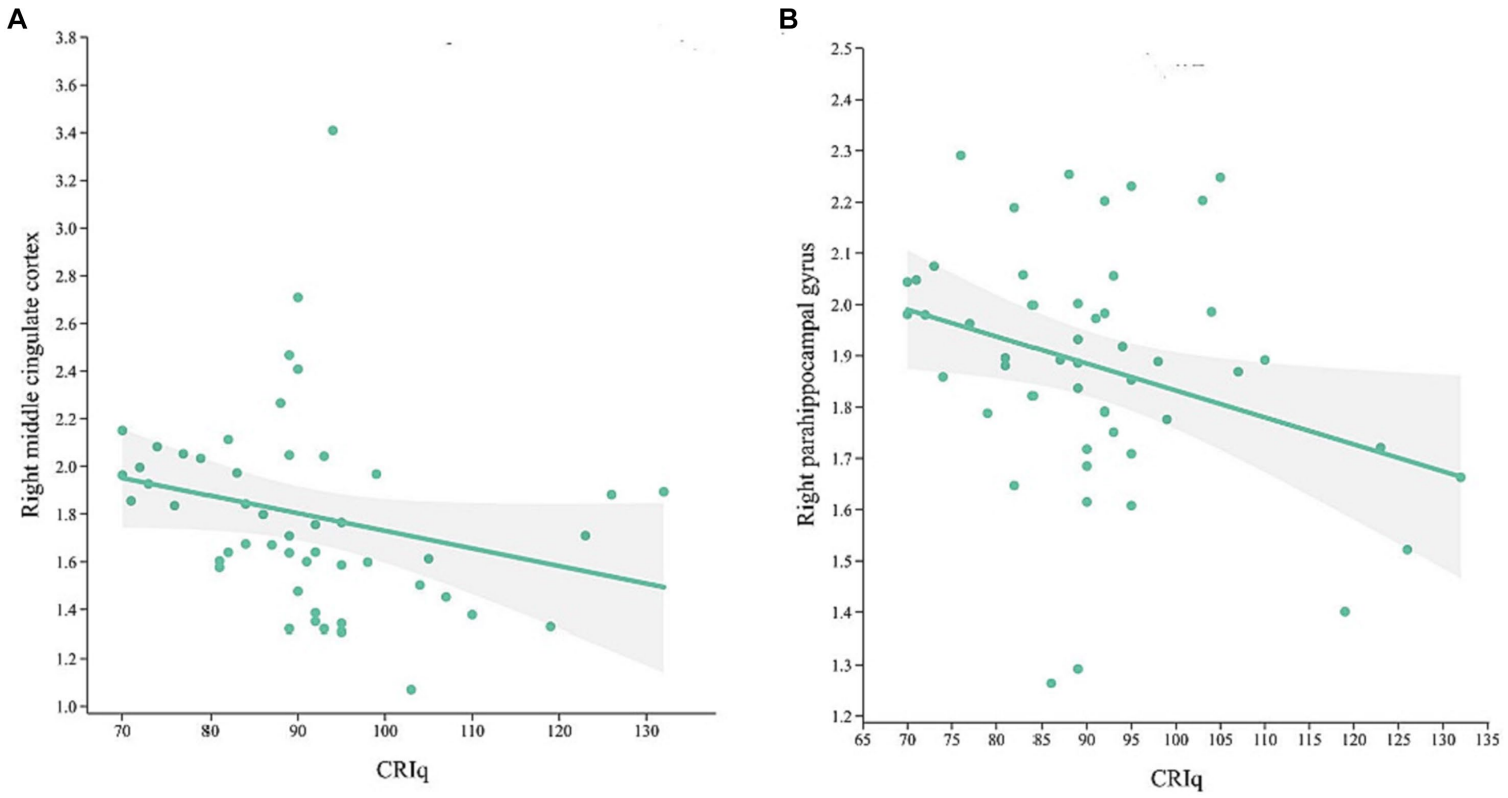
Scatterplots of CRIq and the volume of right middle cingulate cortex **(A)** and right parahippocampal gyrus **(B)** in MCI group.

**Figure 4:**
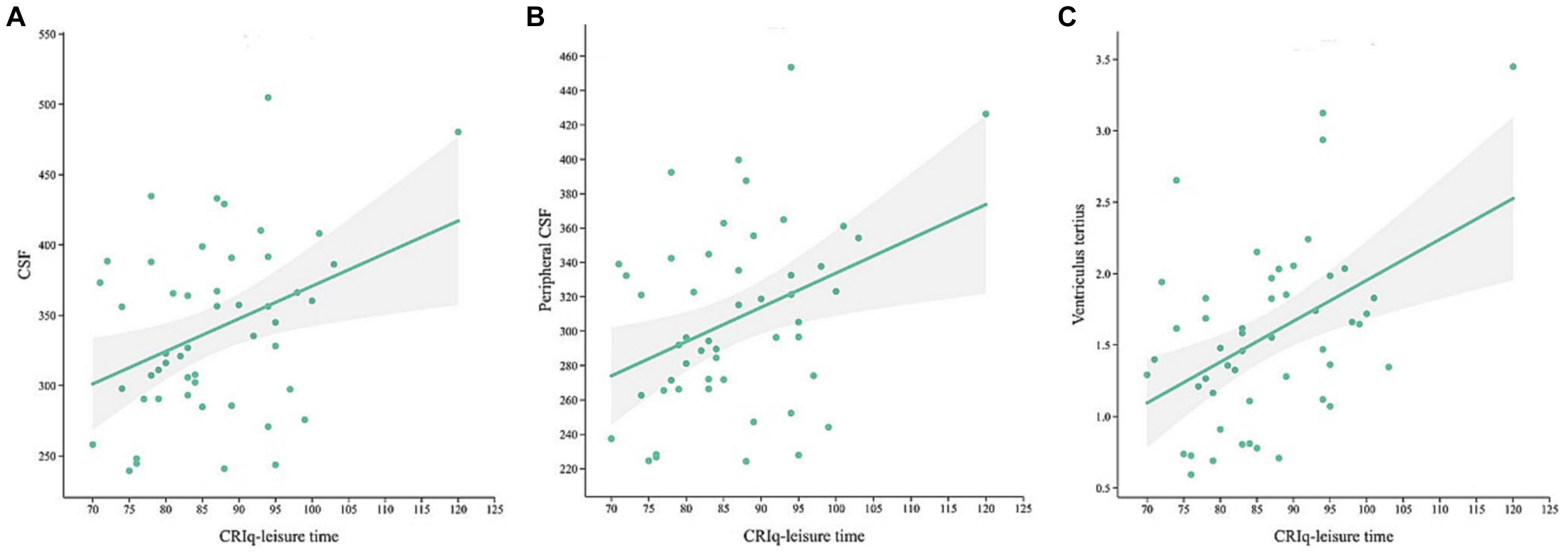
Scatterplots of CRIq-leisure time and the volume of CSF **(A)**, peripheral CSF **(B)** and third ventricle **(C)** in MCI group.

**Figure 5:**
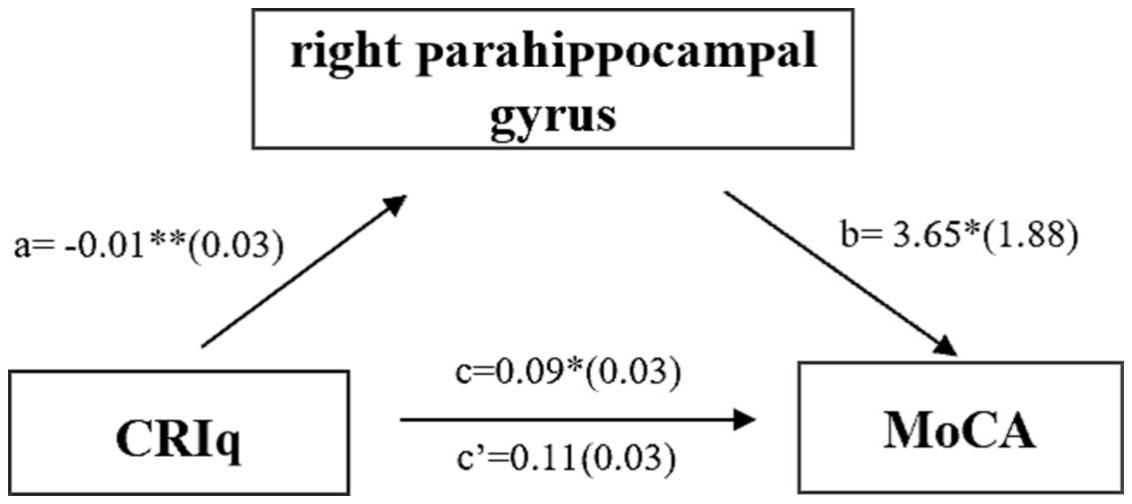
Simple mediation result of the effects of CRIq on the association between right parahippocampal gryus and MoCA score in MCI group; *a*, *b*, *c*, and *c′* are path coefficients representing unstandardized regression weights and standard errors (in parentheses). The *c* path coefficient represents the total effect. The *c′* path coefficient refers to the direct effect. All analyzed *a*, *b*, and *c* paths were significant, ^*^p < 0.05, ^***^p < 0.001.

In MCI group, CRIq was negatively correlated with the volume of the right middle cingulate cortex (Figure 3A, *r* = −0.438, *p* = 0.001) and the right parahippocampal gyrus (Figure 3B, *r* = −0.283, *p* = 0.04). The score of CRIq-leisure time was positively correlated with the volume of CSF (Figure 4A, *r* = 0.367, *p* = 0.009), peripheral CSF (Figure 4B, *r* = 0.364, *p* = 0.009) and third ventricle (Figure 4C, *r* = 0.372, *p* = 0.008). As showed in Figure 5, a significant positive correlation was found between right parahippocampal gryus and MoCA score (path b), while a decrease in CRIq was related to an increase in right parahippocampal gryus (path a) in MCI group. The result of mediation analysis suggested that right parahippocampal gryus mediated the main effect of the relationship between CRIq and MoCA score.

## Discussion

This study found that the CRIq of the HE was significantly higher than that of the MCI group, and was positively correlated with cognitive function scores, further confirming the protective effect of CR on cognitive function. Secondly, we found that MCI patients with higher CR were able to tolerate more severe volume atrophy of the right middle cingulate gyrus and parahippocampal gyrus, suggesting that higher CR could delay the progression of cognitive impairment, and consistent with previous studies (15, 16, 32). The dorsal anterior cingulate cortex subregion is significantly related to sensorimotor network, affective network, and cognitive network, and the middle and posterior cingulate cortex subregion is related to sensorimotor network and perceptual-cognitive system (33). The parahippocampal gyrus is an important region that acts as a connection channel between many cortical and subcortical structures and the hippocampus. It regulates the transformation of short-term memory into long-term memory and plays an important role in the encoding of episodic memory, emotions, spatial position orientation (34). The right middle cingulate gyrus and parahippocampal gyrus are closely related to cognitive function and their morphological and functional changes may cause cognitive function changes, and this study also confirmed that the right middle cingulate gyrus and parahippocampal gyrus are the relevant brain regions of CR in MCI patients. Several reports have shown that a significant negative correlation between whole brain volume (35), left middle cingulate gyrus (18) and CR in MCI patients, and the less educated MCI patients had greater hippocampal volume and less atrophy (36). These results suggest that MCI patients with higher levels of CR correspond to more severe brain pathology at a given level of global cognition, that is, the higher of CRIq, the smaller the volume of gray matter and the more severe the pathological damage, confirming the role of the CR hypothesis in cognitive impairment during the MCI stage of pre-AD.

Another finding is that the volume of CSF, peripheral CSF, third ventricle, and fourth ventricle were higher in MCI group than HE group, and the score of CRIq-leisure time was positively correlated with the volume of CSF, peripheral CSF and third ventricle in MCI group. CSF is produced by the ventricle choroid plexus and enters the subarachnoid space through the aqueduct of midbrain, the intermediate foramen and lateral foramen of the fourth ventrile and the circulation of CSF is essential for the brain to remove wasted molecules. The dysfunction of the ventricle choroid plexus will alter CSF secretion, transport, immunological and barrier functions, which is closely associated with the progression of AD (37). The glymphatic system is also related to the absorption and drainage of CSF, and assist in the circulation of immune cells by draining small molecules from the central nervous system, such as Aβ and tau protein and inflammatory molecules (38). It has been demonstrated that Aβ proteins deposition in the posterior cingulate gyrus, prefrontal, precuneus, and temporoparietal lobes in the brains of MCI patients reduced the functional connectivity between these regions and the rest of the brain, thereby affecting the functional integrity of the default mode network (39). The levels of plasma Aβ and tau proteins were also clearly correlated with the cognitive decline and cerebral atrophy in MCI patients (40). Hence, it could be hypothesized that CR may regulate the increased volume of the ventricular and CSF contributing to increased cerebral blood flow and the rapid flow of CSF, which improves the efficiency of amyloid clearance, as well as may relatively reduce the concentration of Aβ and tau protein in CSF, and thus resist cognitive impairment caused by high concentrations of Aβ and tau protein deposition.

This study confirms the protective effect of CR on MCI patients, which can delay the process of cognitive impairment by improving CR. It suggests that cognitive intervention for cognitive impairment needs to choose a reasonable time window. The use of reasonable prevention of appropriate cognitive training such as more mental activities, more brain exercise, aerobic exercise and so on may be able to control the progression of cognitive decline, reduce the burden of disease caused by cognitive impairment before MCI. However, there was no correlation between CRIq and brain and CSF volume in the HE, possibly due to the ceiling effect in normal people and the insufficient range, which could not to reflect the true level of participants in the HE. Structural MRI has also shown that increased volume of the ventricular system and CSF were associated with increased age to resist the effects of aging (41). In addition, study have found that APOE 4 carriers have undergone significant changes in the gray matter network of the brain as early as middle and young adults (42), indicating that confounding factors may also exist in people with normal cognition, resulting in failure to detect correlations with CR. The present study only studied the effect of CR on the brain and CSF volume of MCI, the CSF biomarkers related to CR are not explored, and the sample size were relatively small. Another potential limitation is the study population was relatively small to form subgroups of participants and analysis effects in subgroups of higher and lower CR. Finally, only correlation analysis was conducted and the lack of adequate research method is a weakness of this study and these results must be interpreted with caution. In future studies, further expansion of sample size and follow-up are needed, as well as studies in terms of CSF biomarkers, and further work is needed to develop reliable analytical methods for analysis effects in subgroups of higher and lower CR.

## Data availability statement

The raw data supporting the conclusions of this article will be made available by the authors, without undue reservation.

## Ethics statement

The studies involving humans were approved by the Ethics Committee of Lanzhou University Second Hospital. The studies were conducted in accordance with the local legislation and institutional requirements. The participants provided their written informed consent to participate in this study.

## Author contributions

LZ: Formal analysis, Investigation, Writing – original draft, Data curation. WY: Data curation, Formal analysis, Investigation, Writing – review & editing. YL: Writing – review & editing. JL: Formal analysis, Software, Writing – review & editing. MZ: Methodology, Software, Writing – review & editing. GL: Project administration, Supervision, Visualization, Writing – review & editing. JZ: Funding acquisition, Project administration, Resources, Supervision, Writing – review & editing.
